# Oxygenation and Hemodynamics during Chest Compressions in a Lamb Model of Perinatal Asphyxia Induced Cardiac Arrest

**DOI:** 10.3390/children6040052

**Published:** 2019-04-03

**Authors:** Munmun Rawat, Praveen Chandrasekharan, Sylvia Gugino, Carmon Koenigsknecht, Justin Helman, Mahdi Alsaleem, Bobby Mathew, Jayasree Nair, Sara Berkelhamer, Payam Vali, Satyan Lakshminrusimha

**Affiliations:** 1 Department of Pediatrics, University at Buffalo, Buffalo, NY 14203, USA; pkchandr@buffalo.edu (P.C.); sfgugino@buffalo.edu (S.G.); carmonko@buffalo.edu (C.K.); jhelman@buffalo.edu (J.H.); mahdials@buffalo.edu (M.A.); bmathew@upa.chob.edu (B.M.); jnair@upa.chob.edu (J.N.); saraberk@buffalo.edu (S.B.); 2 Department of Pediatrics, UC Davis Medical Center, Sacramento, CA 95817, USA; pvali@ucdavis.edu (P.V.); slakshmi@ucdavis.edu (S.L.)

**Keywords:** inspired oxygen, oxygen delivery, chest compression, neonatal resuscitation

## Abstract

The current guidelines recommend the use of 100% O_2_ during resuscitation of a neonate requiring chest compressions (CC). Studies comparing 21% and 100% O_2_ during CC were conducted in postnatal models and have not shown a difference in incidence or timing of return of spontaneous circulation (ROSC). The objective of this study is to evaluate systemic oxygenation and oxygen delivery to the brain during CC in an ovine model of perinatal asphyxial arrest induced by umbilical cord occlusion. Pulseless cardiac arrest was induced by umbilical cord occlusion in 22 lambs. After 5 min of asystole, lambs were resuscitated with 21% O_2_ as per Neonatal Resuscitation Program (NRP) guidelines. At the onset of CC, inspired O_2_ was either increased to 100% O_2_ (*n* = 25) or continued at 21% (*n* = 9). Lambs were ventilated for 30 min post ROSC and FiO_2_ was gradually titrated to achieve preductal SpO_2_ of 85–95%. All lambs achieved ROSC. During CC, PaO_2_ was 21.6 ± 1.6 mm Hg with 21% and 23.9 ± 6.8 mm Hg with 100% O_2_ (*p* = 0.16). Carotid flow was significantly lower during CC (1.2 ± 1.6 mL/kg/min in 21% and 3.2 ± 3.4 mL/kg/min in 100% oxygen) compared to baseline fetal levels (27 ± 9 mL/kg/min). Oxygen delivery to the brain was 0.05 ± 0.06 mL/kg/min in the 21% group and 0.11 ± 0.09 mL/kg/min in the 100% group and was significantly lower than fetal levels (2.1 ± 0.3 mL/kg/min). Immediately after ROSC, lambs ventilated with 100% O_2_ had higher PaO_2_ and pulmonary flow. It was concluded that carotid blood flow, systemic PaO_2_, and oxygen delivery to the brain are very low during chest compressions for cardiac arrest irrespective of 21% or 100% inspired oxygen use during resuscitation.

## 1. Introduction

The majority of newborn infants require limited or no assistance to undergo successful physiologic transition and stabilization at birth. Studies suggest that approximately 10% of infants require some intervention to establish regular respirations at birth with less than 1% needing extensive resuscitative measures such as chest compressions [1]. Newborns who fail to respond to optimized ventilation and chest compressions have a high incidence of mortality and if they survive are at high risk of suffering long-term neurological deficits [2,3]. Initiation of resuscitation with 21% oxygen in term infants is associated with several benefits including earlier time to first cry and reduced mortality [4]. However, the optimal inspired oxygen concentration during chest compressions in neonatal bradycardia and cardiac arrest is controversial. The 2015 International Liaison Committee on Resuscitation (ILCOR) guidelines advocate 100% oxygen when chest compressions are needed [5,6]. This recommendation is based on weak evidence as it is mostly extrapolated from animal, pediatric, and adult literature, as well as based on expert opinion. Owing to poor circulation during chest compressions, pulse oximeters cannot reliably assess oxygenation. Once return of spontaneous circulation (ROSC) is established, pulsed oxyhemoglobin saturation (SpO_2_) can be measured by pulse oximetry and guide supplemental oxygen administration. The infrequent use of chest compressions [7] has impeded the design and completion of rigorous studies to determine the optimal concentration of inspired oxygen during chest compressions in neonatal resuscitation. The previous studies evaluating optimal oxygen concentration during chest compressions were conducted in 1–3 day old piglet models and did not analyze PaO_2_, oxygen content of arterial blood (CaO_2_), or oxygen delivery to the brain during chest compressions [8]. We hypothesized that 100% O_2_ during chest compressions increases oxygen delivery to the brain compared to 21% in a model of perinatal asphyxial cardiac arrest.

## 2. Materials and Methods

This study was approved by the Institutional Animal Care and Use Committee at the State University of New York at Buffalo (Protocol #PED10085N, approved 5 October 2018). Twenty-two time-dated term (139–141-day gestation) pregnant ewes were obtained from May Family Enterprises (Buffalo Mills, PA, USA). Following an overnight fast, the ewes were induced for anesthesia with intravenous diazepam and ketamine. They were intubated with a 10.0 mm-cuffed endotracheal tube (ETT) and ventilated with 21% oxygen and 2–3% isoflurane at 16 breaths/min. The ewes were continuously monitored with a pulse oximeter and an end-tidal CO_2_ (EtCO_2_) monitor [9,10]. Following cesarean section, fetal lambs were partially exteriorized and intubated with a 4.5 mm-cuffed ETT as previously described [11]. The fetal lung fluid in the ETT was drained passively by gravity by tilting the head to the side and, thereafter, the ETT was occluded to prevent gas exchange during gasping in the asphyxial period. Catheters were inserted into the jugular vein (for fluid and medication administration) and right carotid artery (for blood sampling). A 2-mm flow probe (Transonic Systems Inc., Ithaca, NY, USA) was placed around the left carotid artery. A left thoracotomy was performed and a 4-mm flow probe was placed around the left pulmonary artery. The thoracotomy was closed in layers. Electrocardiogram (EKG) leads were attached at the right axilla, left axilla, and right inguinal area (three-lead EKG). The ECG100C (Biopac Systems, Inc., Goleta, CA, USA) was used with Acknowledge Software to record tracings of leads I, II, and III. Following instrumentation, the umbilical cord was occluded and then cut, and the lambs were moved from the maternal abdomen to the radiant warmer. During the asphyxial period (prior to resuscitation), umbilical arterial and low umbilical venous catheters were inserted to measure continuous invasive blood pressures and for epinephrine administration, respectively.

Lambs from three different experimental protocols that followed the Neonatal Resuscitation Program (NRP) algorithm were used: two studies evaluating optimal flush volume with epinephrine and early cord clamping utilized 100% oxygen during chest compressions; one study generating preliminary data for a postnatal hypothermia trial that utilized 21% oxygen. The lambs were grouped based on the O_2_ administered during chest compressions: (1) 21% inspired O_2_ or (2) 100% inspired O_2_. The protocol for cardiac arrest, timing of epinephrine, and dose of epinephrine were similar between the two groups.

A five-minute period of cardiac arrest was observed prior to initiating resuscitation to minimize chances of ROSC with positive pressure ventilation (PPV) alone. Cardiac arrest was defined by the absence of carotid blood flow, arterial blood pressure, and audible heart rate. Resuscitation began by providing PPV with 21% O_2_ by means of a T-piece resuscitator at a rate of 40 breaths/min and initial pressures of 35/5 cm H_2_O. Peak inspiratory pressure (PIP) was adjusted as needed to obtain adequate chest rise. Chest compressions were initiated after 30 s of effective ventilation using a two thumb technique at a 3:1 (chest compression:ventilation) ratio and a depth of one-third anterior–posterior diameter. Upon initiation of chest compressions, inspired O_2_ was continued at 21% or increased to 100% depending on the protocol. The first dose of epinephrine (0.03 mg/kg) was administered if return of spontaneous circulation (ROSC) had not been achieved at 1 min after coordinated chest compressions along with positive pressure ventilation, and every three minutes thereafter until ROSC or for a total of four doses. The ROSC was defined by a heart rate > 60 beats/min with a systolic blood pressure of >30 mm Hg. A baseline arterial blood sample was obtained following instrumentation of the lamb prior to cord occlusion with additional samples at the time of cardiac arrest. Thereafter, blood gases were obtained approximately every minute during resuscitation, at ROSC, and every minute until fifteen minutes following ROSC, followed by every 5 min until 30 min after ROSC. Arterial blood samples were analyzed immediately using a radiometer blood gas analyzer (ABL 800 FLEX, Radiometer A/S, Bronshoj, Denmark).

Following ROSC, the lambs were placed on a ventilator. The PIP and rate were adjusted gradually based on tidal volumes (goal 8–9 mL/kg) and PaCO_2_, and the fraction of inspired oxygen (FiO_2_) was adjusted to maintain pre-ductal saturations between 85–95%. Hemodynamic parameters were continuously monitored. Lambs were euthanized by administering 100 mg/kg pentobarbital sodium (Fatal-Plus Solution; Vortech Pharmaceuticals, Dearborn, MI, USA) at the completion of the study.

### Data Analysis

Arterial blood flow and pressures were continuously recorded using AcqKnowledge Acquisition and Analysis Software (BIOPAC systems, Goleta, CA, USA). Continuous variables were expressed as mean and standard deviation. Oxygen delivery was calculated as oxygen content in the carotid arterial blood (CaO_2_) × carotid blood flow where CaO_2_ = hemoglobin × 1.34 × oxygen saturation of arterial blood (SaO_2_)/100 + 0.0031 × PaO_2_ (in mm Hg).

Categorical variables were analyzed using the chi square test or Fisher’s exact test as appropriate. Continuous parametric variables were analyzed by one-way ANOVA between groups with Fisher’s post hoc test within groups. Non parametric variables were analyzed with the Mann–Whitney U test. Statview 4.0 (SAS Institute, Cary, NC, USA) and XLSTAT (Addinsoft, Long Island City, NY, USA) softwares were used. Statistical significance was defined as *p* < 0.05.

## 3. Results

Twenty-two lambs were asphyxiated to cardiac arrest by umbilical cord occlusion and all lambs achieved ROSC. Out of 22 lambs, 6 were ventilated with 21% inspired O_2_ during chest compressions and 16 lambs were ventilated with 100% inspired O_2_. There were no differences in baseline characteristics (gender distribution, gestational age, birth weight, multiplicity, blood gases, and hemodynamic variables) between the lambs in each group, as is shown in Table 1. Time to asystole was similar in both the groups. Lambs ventilated with 21% O_2_ required 1.4 ± 0.8 doses of epinephrine and those ventilated with 100% O_2_ during CC required 1.3 ± 0.8 doses.

### 3.1. Gas Exchange Parameters

Arterial pH during chest compressions was similar between the two groups—6.85 ± 0.08 in 21% and 6.86 ± 0.08 in 100% inspired O_2_ groups (*p* = 0.8). Following ROSC, in the 21% group, the pH increased to 6.87 ± 0.08 at 5 min and 7.08 ± 0.11 at 30 min whereas in the 100% group the pH was 7.00 ± 0.15 (*p* = 0.07 compared to 21% O_2_ group at 5 min after ROSC) and 7.18 ± 0.15 (*p* = 0.16 compared to 21% O_2_ group at 30 min after ROSC) at 5 and 30 min respectively.

**Figure 1:**
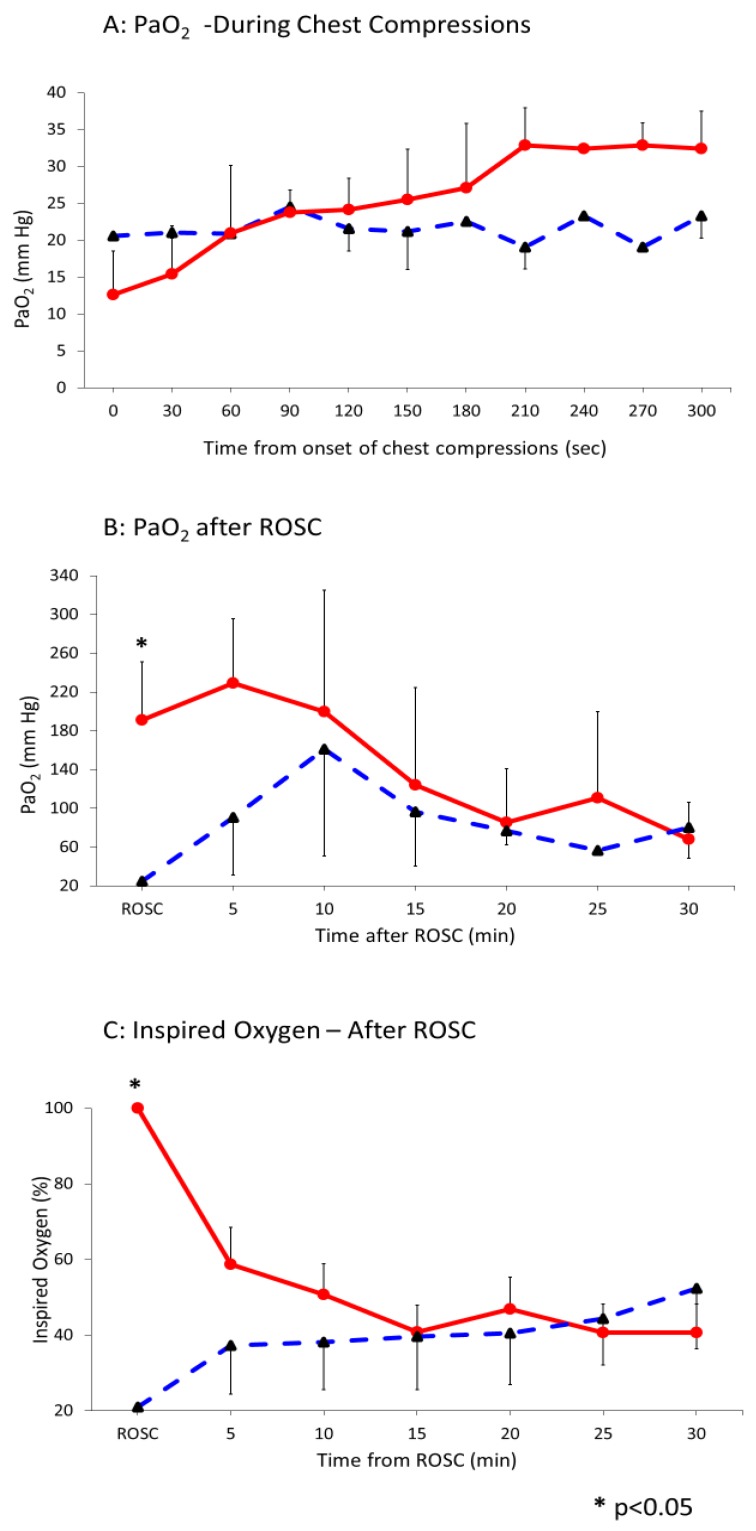
Interrupted line represents 21% and continuous line 100% oxygen. Data presented as average and standard deviation. (**A**) Arterial oxygenation (PaO_2_ mm Hg) during chest compressions (CC). (**B**) Arterial oxygenation (PaO_2_ mm Hg) after return of spontaneous circulation (ROSC). (**C**) The change in fraction of inspired oxygen after ROSC.

The preductal PaO_2_ after asphyxia before starting ventilation was 5.5 ± 2.3 mm Hg. After initiating ventilation with 21% oxygen and chest compressions, the PaO_2_ increased to 21.6 ± 1.6 mm Hg. Following PPV with 100% inspired oxygen, PaO_2_ was 23.9 ± 6.8 mm Hg (*p* = 0.16 compared to 21% O_2_). Figure 1 shows the trend of PaO_2_ in the two groups during chest compressions (Figure 1A) and after ROSC (Figure 1B). Following ROSC, PaO_2_ markedly increased in lambs ventilated with 100% oxygen and gradually reduced with weaning FiO_2_ (Figure 1C). The PaO_2_/ FiO_2_ ratio (P/F) in those lambs decreased from 390 ± 70 at 5 min post ROSC to 170 ± 90 at 30 min. Similarly, in the lambs ventilated with 21% O_2_ the P/F ratio decreased from 240 ± 60 at 5 min to 150 ± 90 at 30 min.

At the time of cardiac arrest, the arterial PaCO_2_ was 125 ± 4mm Hg. It remained similar in both the groups after initiation of PPV followed by chest compressions—113 ± 22 mm Hg in the 21% and 116 ± 21 mm Hg in 100% inspired O_2_ groups (*p* = 0.52). Following ROSC, there was a steady decline in PaCO_2_—86 ± 3 mm Hg and 76 ± 36 mm Hg at 5 min after ROSC (*p* = 0.2) and 49 ± 14 mm Hg and 42 ± 14 mm Hg at 30 min after ROSC (*p* = 0.08) in the 21% and 100% inspired O_2_ groups respectively.

### 3.2. Systemic Hemodynamics

**Figure 2:**
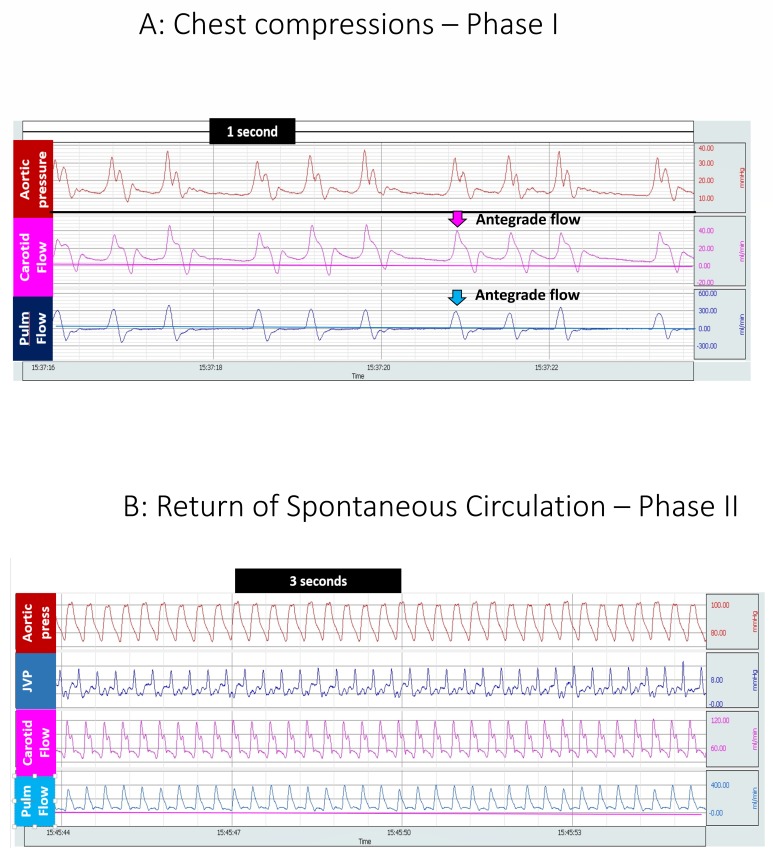
(**A**) BIOPAC snapshot of blood flow during the chest compression; (**B**) BIOPAC snapshot of blood flow after ROSC.

At baseline, mean left carotid arterial blood flow was similar, 28 ± 9 and 27 ± 14 mL/kg/min in the 21% and 100% groups respectively (Table 1). As shown in Figure 2A, during chest compressions, antegrade flow was predominantly observed in the carotid artery during compressions and the mean flows were very low (1.2 ± 1.6 mL/kg/min in 21% and 3.2 ± 3.4 mL/kg/min in 100% oxygen, *p* = 0.07). During the recoil phase of chest compressions, carotid flow was negative. In order to capture the spurts of blood perfusing the brain during compressions, peak carotid flow was calculated and analyzed. Carotid flows increased after achieving ROSC. After ROSC, carotid flow was antegrade both during systole and diastole (Figure 2B).

**Figure 3:**
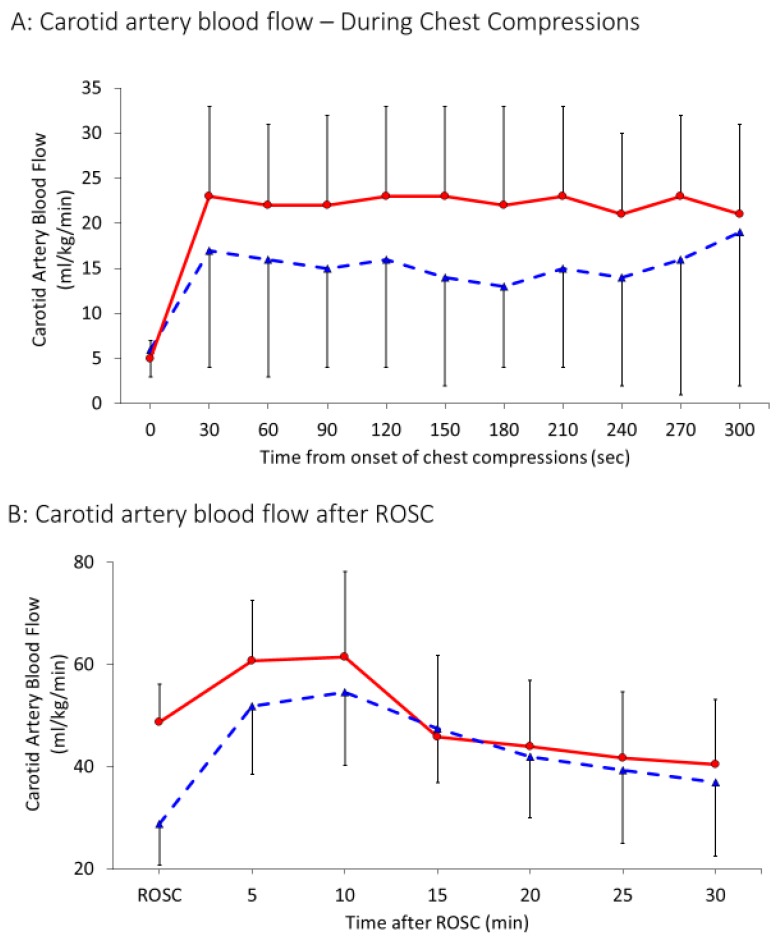
Interrupted line represents 21% and continuous line 100% oxygen. Data presented as average and standard deviation. (**A**) Carotid blood flow in mL/kg/min during CC. (**B**) Carotid blood flow in mL/kg/min after ROSC.

**Figure 4:**
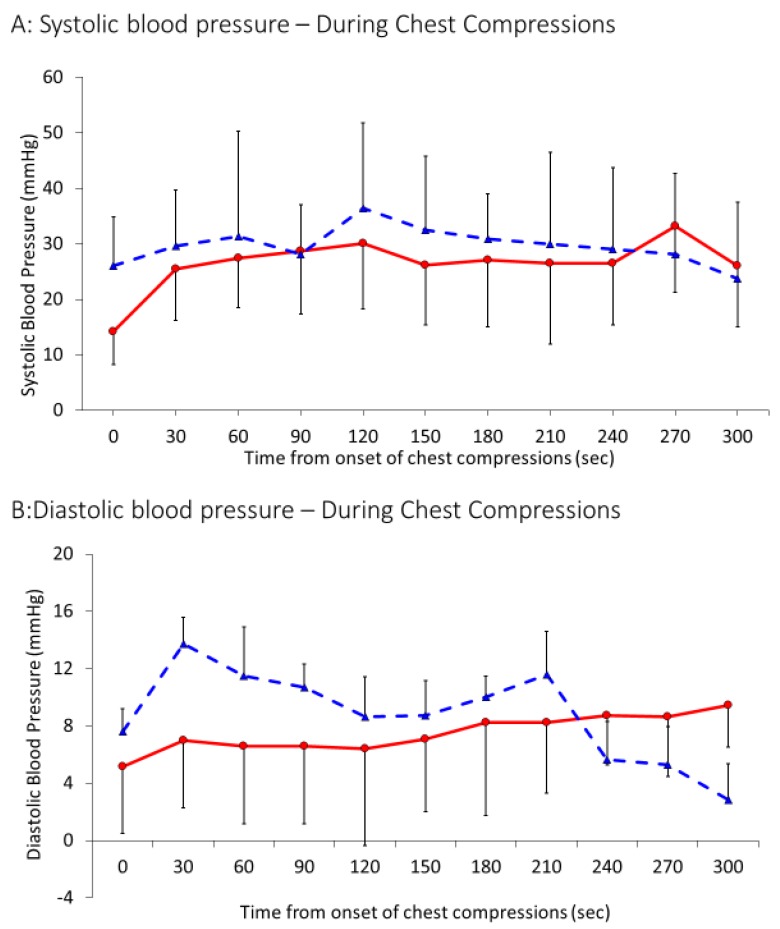
Interrupted line represents 21% and continuous line 100% oxygen. Data presented as average and standard deviation. (**A**) Systolic blood pressure and (**B**) diastolic blood pressure in mm Hg during CC.

Peak values of carotid flow (Figure 3A) during chest compressions were 15 ± 11 mL/kg/min in the 21% group and 21 ± 9 mL/kg/min in the 100% group (*p* = 0.04). After achieving ROSC, maximum systolic carotid flow increased to 43 ± 14 mL/kg/min and 49 ± 17 mL/kg/min in the 21% and 100% oxygen groups respectively as shown in Figure 3B (*p* = 0.16). Systolic blood pressures achieved during chest compressions were similar in both the groups—30 ± 18 mm Hg in the 21% group and 27 ± 11 mm Hg in the 100% group (*p* = 0.11) (Figure 4A). Similarly, diastolic blood pressures during chest compression were similar in the 21% group (9 ± 7 mm Hg) and the 100% group (7 ± 5 mm Hg), *p =* 0.12. Average heart rate after ROSC was also found to be similar in both the groups, 196 ± 30 bpm in the 21% and 205 ± 41 bpm in the 100% group, *p* = 0.2.

### 3.3. Pulmonary Hemodynamics

**Figure 5:**
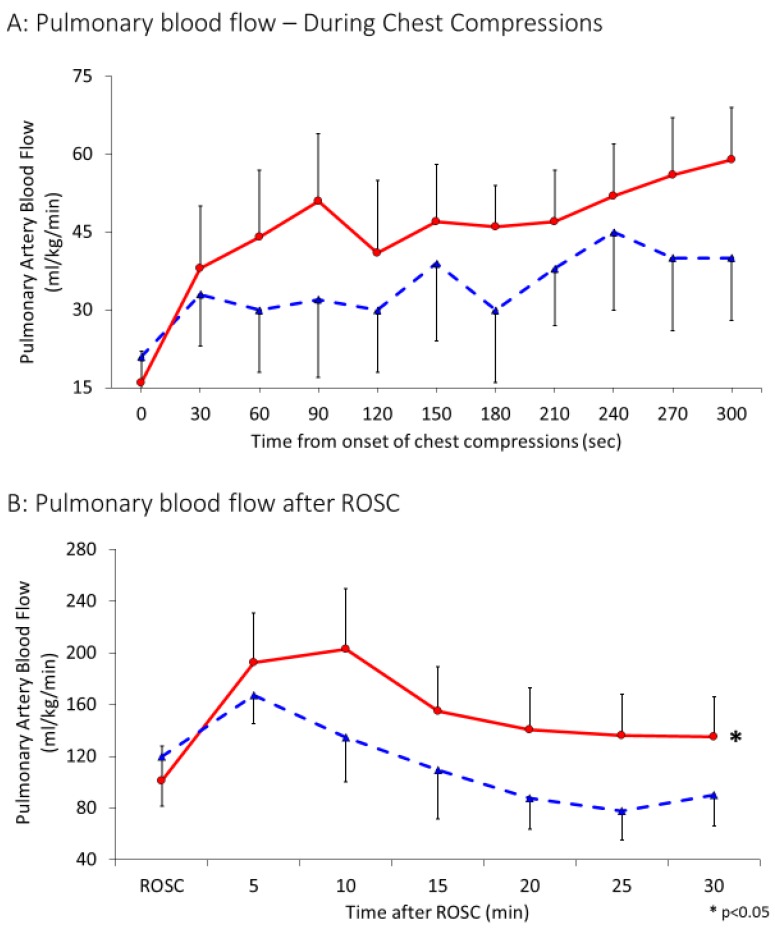
Interrupted line represents 21% and continuous line 100% oxygen. Data presented as average and standard deviation. (**A**) Pulmonary blood flow in mL/kg/min during CC. (**B**) Pulmonary blood flow after ROSC.

Similar to carotid blood flow, antegrade pulmonary blood flow was only observed during the compression phase during chest compressions (Figure 2A). Hence, maximal pulmonary flows during chest compressions were used for analysis. Following initiation of chest compressions, for the first 5 min, left pulmonary arterial blood flow was recorded to be 34 ± 24 mL/kg/min in the 21% group and 45 ± 52 mL/kg/min in the 100% group (*p* = 0.13) (Figure 5 A). As shown in Figure 5B, the flows further increased after achieving ROSC to 112 ± 71 mL/kg/min and 152 ± 138 mL/kg/min in the 21 and 100% O_2_ groups respectively and were significantly different between the two groups (*p* = 0.002).

**Figure 6:**
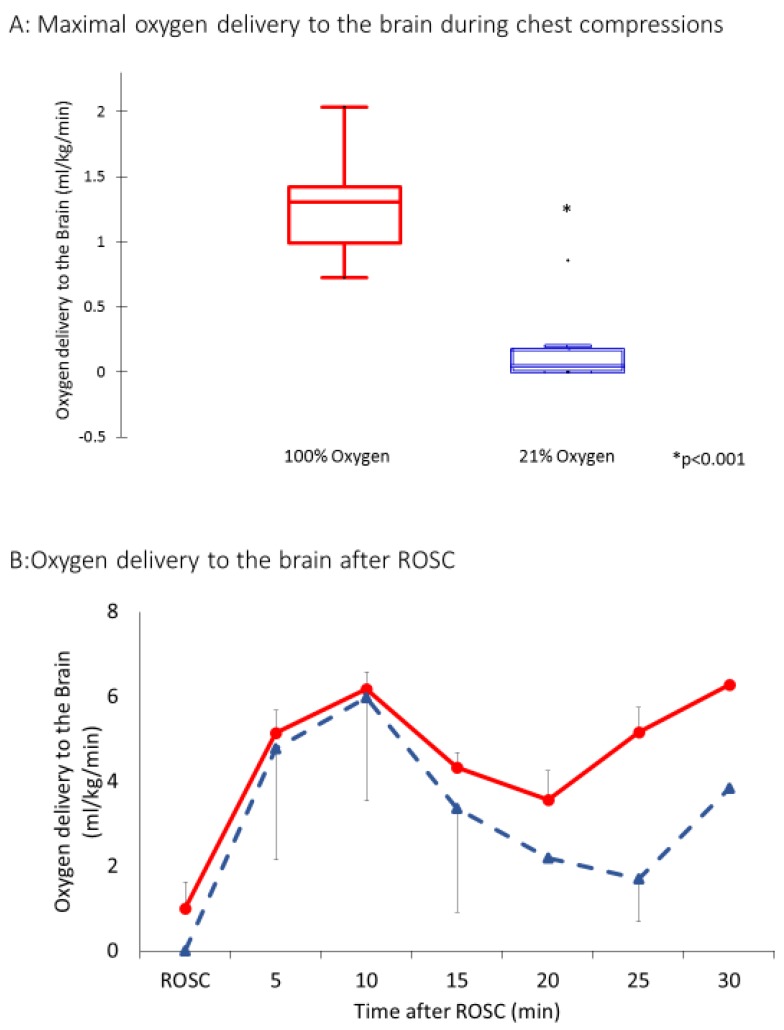
(**A**) Oxygen delivery to the brain during chest compression using the antegrade systolic carotid blood flow represented as a bar graph. (**B**) Oxygen delivery to the brain after return of spontaneous circulation. Interrupted line represents 21% and continuous line 100% oxygen. Data presented as average and standard deviation.

Oxygen delivery to the brain during chest compressions and after ROSC are shown in Figure 6A,B. During the baseline fetal period, oxygen delivery to the brain was 2.1 ± 0.3 mL/kg/min. During chest compressions, oxygen delivery was 0.05 ± 0.06 mL/kg/min in the 21% group and 0.11 ± 0.09 mL/kg/min in the 100% group. It improved in both groups after ROSC as expected but was significantly higher in the 100% inspired oxygen group (3 ± 3 mL/kg/min in the 21% group and 5 ± 2 mL/kg/min in the 100% group).

Since carotid blood flow occurred in short spurts during the compression phase only, we calculated the oxygen delivery during the peak carotid flow during chest compressions. The peak oxygen delivery was not normally distributed. The oxygen delivery to the brain at the peak of chest compressions with 21% oxygen was 0.075 mL/kg/min (IQR: 0–0.2) and was significantly lower than with 100% oxygen 1.4 mL/kg/min (0.9–1.25, *p* < 0.0001).

## 4. Discussion

A primary contributor to death and disability in patients of all ages undergoing resuscitation is anoxic brain injury that typically follows a severe ischemia/reperfusion insult. Hypoxic ischemic encephalopathy can occur among newly born infants following perinatal asphyxia and resuscitation in the delivery room. Birth asphyxia accounts for about 23% of the approximately 2.5 million neonatal deaths each year worldwide [12,13]. Severe asphyxia is associated with bradycardia and cardiac arrest necessitating chest compressions to maintain circulation. Maintaining perfusion and oxygenation of the brain is crucial to improve outcomes following arrest. We evaluated the effect of supplemental oxygen on hemodynamics, gas exchange, and oxygen delivery to the brain during chest compressions for perinatal asphyxia cardiac arrest. We did not find any difference in carotid blood flow, pulmonary blood flow, PaO2, or mean oxygen delivery to the brain between 21% and 100% inspired oxygen during chest compressions for perinatal arrest. Oxygen delivery to the brain at the peak of chest compressions was significantly higher with 100% oxygen but the clinical significance of this finding is unknown.

There are minimal clinical data comparing 21% and 100% oxygen during neonatal chest compressions in the delivery room. A systematic review of 10 clinical studies published in 2008 comparing 21% and 100% oxygen for resuscitation of term newborns (*n* = 2133) showed reduced mortality with 21% oxygen (8.2% vs. 12.9%) in neonates [4]. However, during advanced stages of resuscitation, some infants enrolled in the 21% oxygen group were switched to higher levels of inspired oxygen. For example, two of the largest trials in this meta-analysis used different techniques in severely asphyxiated infants not responding to resuscitation. Saugstad et al. increased inspired oxygen to 100% if the infant did not respond adequately to resuscitation within 90 s after birth [14]. Vento et al. evaluated 30 severely asphyxiated infants (0.2% of all deliveries). [15]. Severe asphyxia was defined as Apgar score < 3 at one minute, cord arterial pH < 7, heart rate < 60/min at 1 min, hypotonia, apnea, and/or Apgar score < 5 at 5 min. Among these severely asphyxiated infants, 1/16 assigned to 21% and 6/14 assigned to 100% oxygen died. However, it is not clear how many of these infants were in cardiac arrest requiring chest compressions. Hence, there are no clinical data evaluating oxygenation and hemodynamics during chest compressions for cardiac arrest in newly born infants.

Several studies have investigated supplemental oxygen during chest compressions in postnatal piglet models. Linner et al. studied one-day old piglets with apnea-induced cardiac arrest. Cardiac arrest was defined as heart rate < 50/min with mean systemic blood pressure < 25 mm Hg [8]. There was no statistical difference in time to reach ROSC (defined as heart rate > 150/min) with recovery at 67 (60–76) s with 21% oxygen and 88 (76–126) s with 100% oxygen. No blood gases were performed during the early phases of resuscitation in this study and the first blood gas was reported at 120 s after onset of resuscitation when the majority of piglets had already achieved ROSC. The authors reported high brain tissue PO_2_ with 100% oxygen resuscitation. Solevag et al. studied 12–36 h old piglets with apnea induced arrest and resuscitated them with 21% and 100% oxygen [16]. Median time to ROSC was similar between the two groups, reported as 150 (115–180) s with 21% oxygen and 135 (113–168) s with 100% oxygen. No blood gases or oxygen saturation results were provided with these studies, however oxygen saturation levels were higher after ROSC in the 100% oxygen group. Solevag et al. repeated a study in 1–3 day old piglets with cardiac arrest defined as absence of audible heart beat and demonstrated no difference in time to ROSC with 21% or 100% oxygen but a higher left ventricular oxidized glutathione (GSSG/GSH) ratio in 100% oxygen group [17]. These translational studies have identified a paradox relative to oxygen delivery to the injured brain but have mainly focused on the post-ROSC phase [8,16]. A recent meta-analysis evaluating 100% oxygen during chest compressions reviewed 8 studies involving 323 animals (and included the abstract based on the current study) [18,19]. There was no difference in mortality or time to ROSC. None of these other studies evaluated oxygen delivery and hemodynamics during chest compressions. In addition, six out of seven studies used newborn piglet models that had already transitioned to extrauterine life and oxygen was not titrated based on NRP recommended oxygen saturation ranges after achieving ROSC. A transitioning newborn infant has fluid-filled lungs, a patent ductus arteriosus, and increased pulmonary vascular resistance as compared to a 1–2 day old infant who has completed perinatal cardiopulmonary transition. Perez-de-Sa et al. evaluated the only other study using lambs and reported that immediately after ROSC, lambs had higher brain tissue oxygen levels with 100% oxygen resuscitation [19].

We have developed a transitional model of prolonged asphyxiated lamb that best mimics an infant with severe perinatal asphyxia [11]. We have previously shown in this model that asphyxia by umbilical cord occlusion resulted in a significant decrease in arterial pH (6.9 ± 0.01) and preductal SpO_2_ (38 ± 2) compared to normal term fetal lambs (pH—7.3 ± 0.09 and SpO_2_—53 ± 1.4%) [20]. Contrary to our hypothesis, PPV with 100% oxygen during chest compressions for cardiac arrest did not significantly improve systemic oxygenation or oxygen delivery. The mean PaO_2_ during chest compressions after cardiac arrest was 20 mm Hg as compared to 40–60 mm Hg in a term newborn infant. We speculate that although alveolar PAO_2_ is increased by 100% inspired oxygen, low pulmonary blood flow results in poor gas exchange and low PaO_2_. An interesting observation was that oxygenation during spurts of antegrade flow in the carotid artery during chest compressions was better with 100% oxygen. The precise benefit of such intermittent perfusion with oxygenated blood and its role in preventing hypoxic injury to the brain is not known. However, after ROSC, the carotid flow increases resulting in hyperoxemia with 100% O_2_. We speculate this may lead to increased oxygen delivery to the brain which may potentially cause reperfusion injury to the brain. We suggest that inspired O_2_ concentration should be weaned to 21–30% oxygen immediately after ROSC and titrated based on preductal pulse oximetry with a target goal of 85–95%.

We acknowledge several limitations in our study. This study was not a randomized trial and the data were extracted and analyzed from lambs studied for other protocols. The oxygen concentration was not masked. However, the systolic pressures generated during chest compressions were similar between the two groups suggesting that the intensity of chest compressions were similar between the two groups. This was a model of complete cardiac arrest and response to 100% oxygen may be different with less profound asphyxia with bradycardia (heart rate < 60/min but not 0/min) necessitating chest compressions. We did not evaluate brain and lung tissue for evidence of oxidative stress or hypoxic-ischemic injury. Finally, response to 100% O_2_ may be different in postnatal arrest after establishment of lungs as the site of gas exchange as compared to our perinatal asphyxia model. Despite these limitations, we provide comprehensive oxygenation and hemodynamic data during chest compressions in perinatal arrest.

## 5. Clinical Implications

Our results demonstrate very low pulmonary and carotid flows during chest compressions for cardiac arrest. In fact, it appears blood flow is intermittent to these essential organs. The preductal PaO_2_ values are low and similar to fetal baseline levels and did not differ between 21% and 100% oxygen groups, probably secondary to low pulmonary blood flow. We did not demonstrate a difference in oxygen delivery to the brain with 21% and 100% oxygen. These findings neither support nor refute current recommendations to provide supplemental 100% oxygen during chest compressions.

However, following ROSC, lambs that received 100% oxygen had post-asphyxial increased carotid blood flow and hyperoxia in spite of rapid weaning of FiO_2_ over the first 5 min after ROSC. Further studies evaluating abrupt decrease in inspired oxygen concentration to 21–30% immediately after achieving ROSC need to be evaluated to minimize the risk of hyperoxic reperfusion injury to the brain.

## 6. Conclusions

Systemic and pulmonary hypoperfusion is common during chest compressions in cardiac arrest and brain oxygen delivery is extremely low irrespective of the concentration of inspired oxygen levels. Future randomized and masked studies that compare low vs. high supplemental oxygen during newborn resuscitation with chest compressions for cardiac arrest and bradycardia are needed to assess markers of oxidative stress, as well as assess immunohistochemical changes in the brain to provide a better understanding on the optimal oxygen therapy during resuscitation of newborns.

## Figures and Tables

**Table 1:** Baseline characteristics prior to onset of positive pressure ventilation and chest compressions.

Inspired O_2_ Concentration during Chest Compressions	21% Oxygen (*n* = 6)	100% Oxygen (*n* = 16)
Male (%)	4 (66%)	7 (44%)
Gestational age, days (term = 147)	141 ± 1	141 ± 1
Birth weight, kg	3.73 ± 0.73	3.7 ± 1.09
Multiple gestation, *n* (%)	3 twins (50%)	8 twins + 3 triplets (69%)
Baseline arterial pH—after instrumentation	7.20 ± 0.2	7. 18 ± 0.1
Arterial pH at onset of resuscitation	6.85 ± 0.09	6.86 ± 0.07
Partial pressure of oxygen in arterial blood (PaO_2_ mm Hg)	22 ± 4	19 ± 7
Mean pulmonary artery blood flow (mL/kg/min)	8 ± 13	12 ± 18
Mean carotid artery blood flow (mL/kg/min)	28 ± 9	27 ± 14
Time to asystole (s)	637 ± 197	630 ± 333

Note: The baseline characteristics were not significantly different.
